# Effects of dietary calcium on atherosclerosis, aortic calcification, and icterus in rabbits fed a supplemental cholesterol diet

**DOI:** 10.1186/1476-511X-5-16

**Published:** 2006-06-23

**Authors:** Howard HT Hsu, Nathan C Culley

**Affiliations:** 1 Department of Pathology and Laboratory Medicine, Cardiovascular Research Division, University of Kansas Medical Center, Kansas City, KS, 66160, USA; 2 Laboratory Animal Resources, University of Kansas Medical Center, Kansas City, KS, 66160, USA

## Abstract

**Background:**

Vascular calcification is implicated in myocardial infarction, instability and rigidity of the aortic wall, and bioprosthetic failures. Although an increase in the calcium (Ca) content in atherogenic diets has been shown to decrease atherosclerosis in rabbits, whether Ca supplementation and deficiency can affect atherosclerosis-related aortic calcification remains unknown.

**Results:**

New Zealand White male rabbit littermates were fed an atherogenic diet containing 0.5% cholesterol and 2% peanut oil. The Ca content of the diet, which normally contains 1%, was adjusted to 0.5 or 3%. Segments of thoracic aortas were dissected from rabbits for histological evaluations and Ca and Pi determinations. Rabbits with calcium supplementation were maintained for 4 months, whereas those with calcium deficiency were maintained for 2 1/2 months due to severe icterus beyond this stage. The ratios of intimal to medial areas and calcified to intimal areas were used to semi-quantify lesion accumulation and calcification, respectively. Icterus was estimated from the extent of yellowing of the skin, sclera, and mucous membranes along with gross evidence of hepatic lipidosis and/or biliary obstructions. Statistical analysis of 16 matched littermates shows that Ca supplementation significantly decreased the lesions by 41% (p < 0.05) and markedly inhibited calcification by 62% (p < 0.05). Statistical analysis of 11 matched littermates shows that Ca deficiency significantly increased the lesions by 2.7-fold (p < 0.05) and that the diet caused a small but significant calcification not seen in the sibling groups with normal dietary Ca. Ca supplementation caused a significant 30% decrease in serum cholesterol (p < 0.05). Calcium deficiency increased serum cholesterol by 57% (p < 0.001). Serum cholesterol and LDL-cholesterol levels in Ca deficient rabbits were 2-fold higher than those with high Ca diets. Ca supplementation decreased soluble Ca and Pi content in aortas, suggesting that this effect may underlie the effects of Ca supplementation on calcification. Calcium deficiency increased icterus by 33% (p < 0.05), which may affect hepatic clearance of cholesterol, while calcium supplementation decreased it by 43% (p < 0.001).

**Conclusion:**

Ca supplementation to an atherogenic diet inhibits atherosclerosis, aortic calcification, and icterus, whereas a Ca deficient-diet promotes them.

## Background

Increased Ca content in diets supplemented with cholesterol has been shown to decrease atherosclerosis in rabbits [1,2]. Two epidemiological studies suggested that high levels of Ca in drinking water may decrease atherosclerosis [3,4]. However, whether the increase in Ca intake could also affect aortic calcification through altered levels of Ca × P ion products in aortas was unclear. It is equally possible that Ca supplementation or deficiency could affect the calcification through its primary effect on atherosclerosis. Since vascular calcification is implicated in myocardial infarction [5], instability and rigidity of the aortic wall [6], and bioprosthetic failures [7], it is imperative to study the effect of dietary Ca on aortic calcification.

During the course of this study, we also found that cholesterol diets often caused increased evidence of icterus from liver impairment, which means the diets may impede the ability of liver to metabolize lipids, thereby influencing serum cholesterol levels. In this report, we sought to determine whether dietary Ca also affects icterus associated with supplemental cholesterol diets. We herein show that Ca supplementation decreases while deficiency increases lesion formation and aortic calcification in rabbit littermates. For the first time, we demonstrate that dietary Ca deficiency increases the chance of developing signs of icterus, while calcium supplementation decreases the chance of developing icterus in rabbits receiving the atherogenic diet.

## Results and discussion

### Effects of calcium supplementation on atherosclerosis and aortic calcification

**Figure 1:**
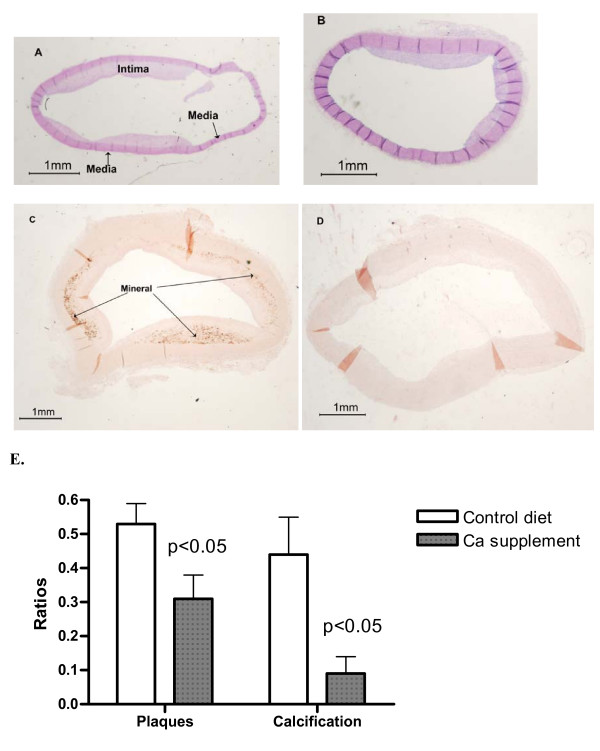
Effects of Ca supplementation for 4 months on the accumulation of plaques and minerals in aortas. Panels A–D show typical examples of the effects of calcium supplementation on accumulation of lesions and calcification in a sibling group. The rationale for the selection of the middle and proximal sections, respectively, for lesion accumulation and calcification was given in detail in the Experimental Procedures section. The cross sections of the middle section of aortas from sibling brothers were stained with H&E (Panels A and B) to show intimal thickening (lesions). The cross sections of the proximal section were stained with AR (Panels C and D) to show mineralization (arrows). A & C are cross sections obtained from rabbits fed the atherogenic diet with normal levels of dietary Ca. B and D are cross sections from rabbits with Ca supplementation. The thickened intima to media ratio was used to semi-quantify the extent of lesion accumulation as described in "Experimental Procedures" section. Likewise, the extent of calcification was expressed as the ratio of calcified area to the area of thickened intima. Intima/media ratios: A, 0.77 vs. B, 0.32. Calcified area/intima ratio: C, 0.47 vs. D, 0.00. Panel E provides the paired Student's *t*-test for the significance of the differences in the ratios between the two respective groups each with 16 siblings.

We previously demonstrated that cholesterol supplementation induced aortic calcification in the lower zone of lesions (LZP) adjacent to the media progressing along LZP [8,9]. After 4 months of the atherogenic diet, gross examinations of the thoracic aortas indicated that the proximal arch was fully covered with lesions. In contrast, about half of the middle sections (2.5 cm from the arch joint) were covered with the lesions. At this stage, about half of the lesions in the proximal sections were filled with mineral deposits identifiable with alizarin red stains (AR), whereas the distal sections were rarely calcified. Thus, the selection of the middle and proximal sections for the quantification of lesions and mineral accumulation, respectively, was preferable for statistical analysis. These effects on lesion formation and calcification were semi-quantified using relative ratios of the intimal to medial areas and the calcified to intimal areas, respectively, as described in the "Experimental Procedure" section. Fig. 1A & B show a typical example of the inhibitory effect of calcium supplementation for 4 months on the accumulation of lesions within a sibling group. A rabbit sibling with normal levels of Ca in the atherogenic diet exhibited a 0.77 ratio of the intima to media (Fig. 1A) vs. a 0.32 ratio from another sibling with Ca supplementation (Fig. 1B). Fig. 1C & D show the effect of calcium supplementation on calcification. The ratios of calcified areas to the lesions were 0.47 and 0, respectively, for a sibling with normal Ca diets and the other with calcium supplementation. Statistical analysis of 16 matched littermates shows that Ca supplementation for 4 months significantly decreased the lesions by 41% (p < 0.05) and markedly inhibited calcification by 62% (p < 0.05) (Fig. 1E). The control rabbits fed normal chow for 4 months developed neither lesions nor aortic calcification (not shown). The effect of calcium supplementation on lesion formation is consistent with the observation by Yacowitz et al. [1] and Renaud et al. [2]. For the first time, we demonstrate that calcium supplementation inhibits aortic calcification. Since aortic calcification visible by AR staining did not occur until the lesions were well developed [8,9], it is likely that calcium effects could be a consequence of the primary effect of supplemental Ca on atherosclerosis.

### Effects of dietary calcium deficiency on atherosclerosis and aortic calcification

**Figure 2:**
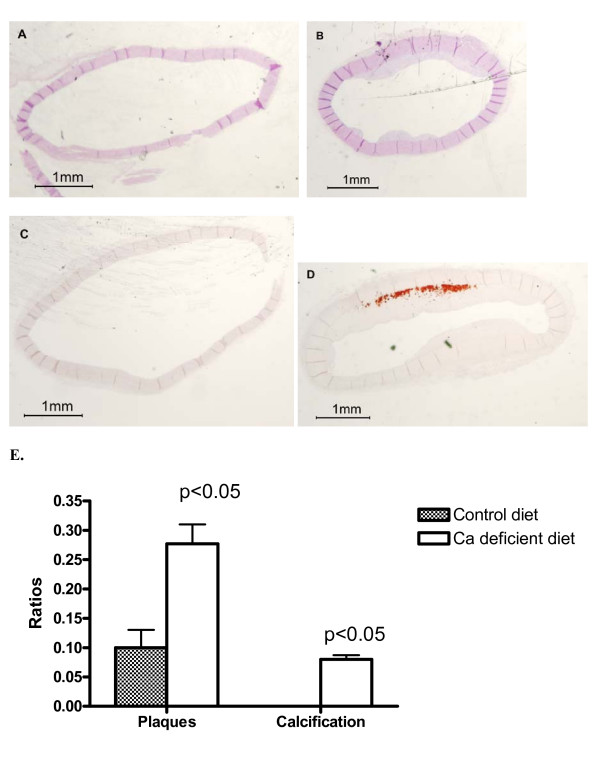
Effects of the calcium deficient diet for 2 1/2 months on the development of lesions and calcification in sibling rabbits. The cross sections of the middle section of aortas were stained with H&E (Panels A and B) and the proximal section with AR (Panel C and D). Panels A and C: Cross-sections obtained from a sibling fed the atherogenic diet with normal levels of Ca. Panels B and D: The sections from a sibling fed the atherogenic diet with calcium deficiency. The semi-quantification for intimal thickening and calcification was described in Fig. 1 legend. Panel E provides the paired Student's *t*-test for the significance of the differences in these ratios between the two respective groups each with 11 siblings. Note that the graphic bar representing calcification for normal dietary Ca groups was not seen since there was no calcification in this group during 2 1/2 months of cholesterol intervention.

Fig. 2 shows the effect of dietary calcium deficiency for 2 1/2 months on accumulation of lesions and calcification in a sibling group. The Ca deficiency study was not extended beyond this time period since rabbits barely survived due to severe liver impairment and subsequent icterus. The effect was semi-quantified using the ratios of intima thickening/media for lesion formation and calcified area/intima for mineral deposition. A rabbit sibling with normal levels of dietary Ca in the atherogenic diet exhibited neither lesions in the cross section of the middle part of aortas (Fig. 2A) nor mineral deposits in the proximal part (Fig. 2C). In contrast, a sibling with the calcium deficient diet shows formation of lesions with an intima/media ratio of 0.28 (Fig. 2B) and calcified area/intima ratio of 0.12 (Fig. 2D). Statistical analysis of 11 matched littermates shows that Ca deficiency significantly increased the lesions by 2.7-fold (p < 0.05) and that the diet caused a small but significant calcification not seen in the sibling groups with normal dietary Ca (Fig. 2E). The effect of the Ca deficient diet on atherosclerosis is consistent with the observations of Yacowitz et al. [1] and Renaud et al. [2]. For the first time, we demonstrate that calcium deficiency promotes aortic calcification.

### Effects of dietary Ca on serum lipids in rabbits fed high cholesterol diets

**Figure 3:**
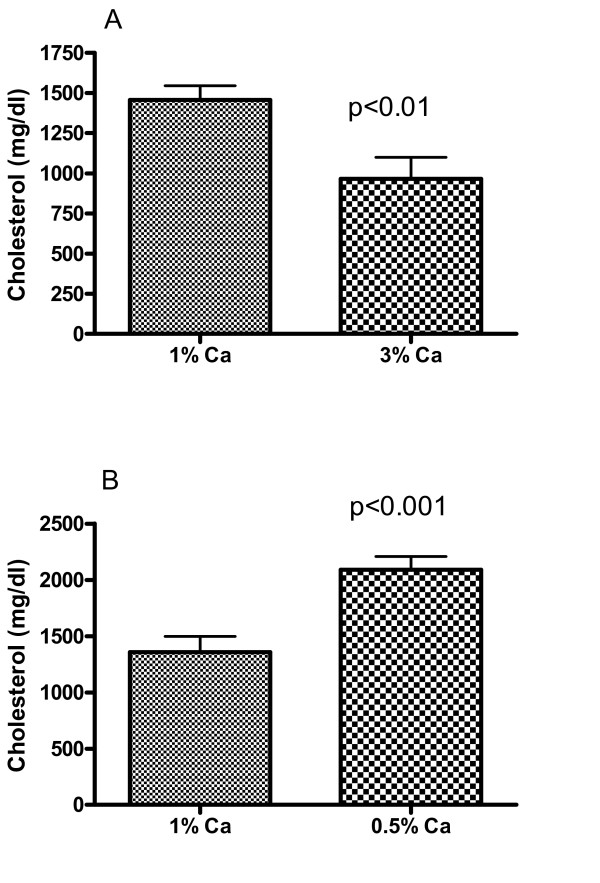
Effects of dietary Ca on serum cholesterol. The total serum cholesterol was measured using a commercial kit (Thermo DMA). Panel A shows the effect of calcium supplementation (3% Ca) vs. the control group (1% Ca) for 4 months. Panel B shows the effect of calcium deficiency (0.5% Ca) for 2 1/2 months. There were 11 matched littermates in each group.

**Figure 4:**
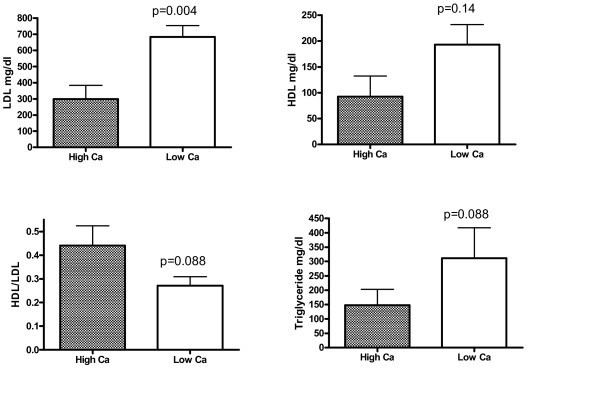
Effects of Ca deficiency on serum levels of lipid risk factors. Since the serum levels of HDL-cholesterols and triglyceride in calcium deficient group were insignificantly different from the normal Ca group (p > 0.05), a comparison between calcium deficiency and supplementation was used to better see the Ca effects. The LDL- and HDL-cholesterol and triglyceride from diluted serum samples were measured using an automation system in a clinical laboratory. There were 11 matched littermates between high and low calcium groups.

To determine whether dietary Ca may affect levels of various lipid risk factors in the serum and thereby influence atherosclerosis and calcification, we measured the serum lipids from rabbits treated with various Ca diets. Consistent with those findings by Yacowitz et al. [1,10], Ca supplementation caused a significant 30% decrease in serum cholesterol (p < 0.05, Fig. 3). Calcium deficiency increased serum cholesterol by 57% (p < 0.001). Except for LDL cholesterol, which was significantly reduced by calcium supplementation and increased by calcium deficiency (data not shown), HDL-cholesterol and triglyceride were not significantly affected by Ca diets. To further support this conclusion, we compared the difference between calcium supplementation and deficiency on these two parameters. As shown in Fig. 4, serum cholesterol and LDL-C levels in Ca deficient rabbits were 2-fold higher than those with high Ca diets. Although HDL, HDL/LDL ratios, and triglyceride seemed different between these two groups, the effects were statistically insignificant (p > 0.05). A further statistical analysis revealed the Power levels of 30–40% at α <0.05, indicating that it is unlikely that Ca deficiency affected these parameters.

### Effect of dietary Ca on icterus development in rabbits fed high cholesterol diets

**Figure 5:**
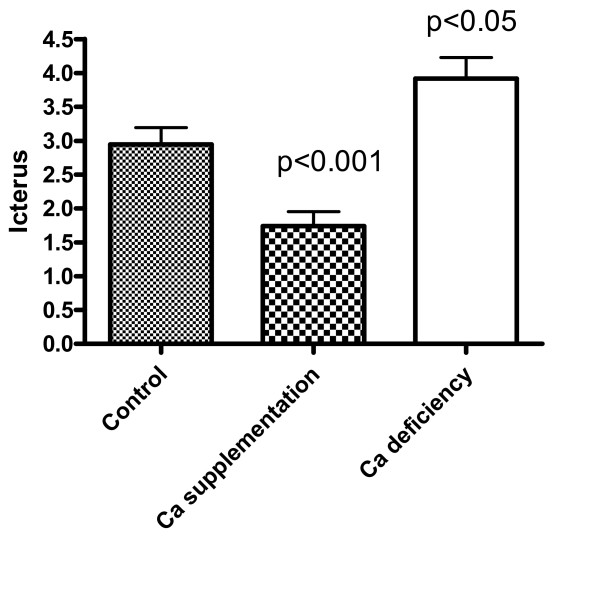
Effects of Ca supplementation and deficiency on icterus. Icterus was diagnosed by the extent of yellowing of the skin, sclera, and mucous membranes along with gross evidence of hepatic lipidosis. A scale of 5 was arbitrarily given using the combined signs of icterus to indicate the relative degree of icterus. There were 11 matched littermates among 3 groups with various dietary Ca levels.

During the course of this investigation, we often found cholesterol supplementation caused icterus in the rabbits. Since icterus may be a result of liver impairment which can affect hepatic clearance of lipids, thereby altering serum lipid levels, the effects of Ca on the development of icterus were examined. As shown in Fig. 5, calcium deficiency increased icterus by 33% (p < 0.05) while calcium supplementation decreased it by 43% (p < 0.001).

### Effect of dietary Ca on serum Ca

**Figure 6:**
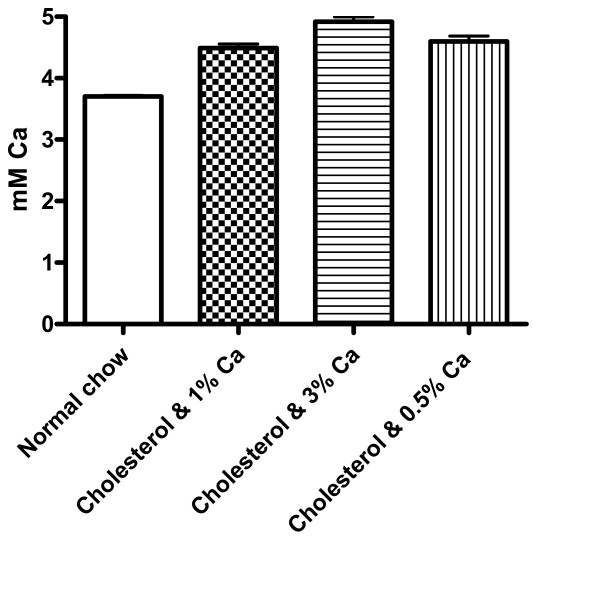
Effects of cholesterol and Ca diets on serum Ca. One-Way variance analysis was used to evaluate differences among groups. Cholesterol diet caused a significant higher serum Ca than the control group (p < 0.01). There were no differences among atherogenic diet groups with various levels of Ca.

The mechanism of dietary Ca on atherosclerosis and aortic calcification is difficult to elucidate since Ca has diverse effects on a variety of cells. It is conceivable that dietary Ca in atherogenic diets could affect the serum Ca levels. The changes in the serum Ca may then affect tissue level of Ca, thereby affecting lipid metabolism or cellular vulnerability to cholesterol. As shown in Fig. 6, cholesterol supplementation significantly increased serum Ca levels from a normal level of 3.70 ± 0.57 mM (10) to 4.49 ± 0.69 (10), representing a 20% increase (p < 0.001). Ca levels in sera from rabbits with Ca supplementation and deficiency were 4.92 ± 0.27 (11) and 4.60 ± 0.29 (11), respectively. The differences between these two groups were statistically insignificant (p > 0.05). The effect of Ca diets on serum Pi was statistically insignificant likely due to the large fluctuations in the serum phosphate content in contrast to serum Ca (data not shown). The pathological relevance of increased levels of serum Ca as a result of cholesterol feeding was unclear as was the ineffectiveness of dietary Ca to alter serum Ca levels.

### Effect of dietary Ca on the accumulation of soluble Ca in aortas

**Figure 7:**
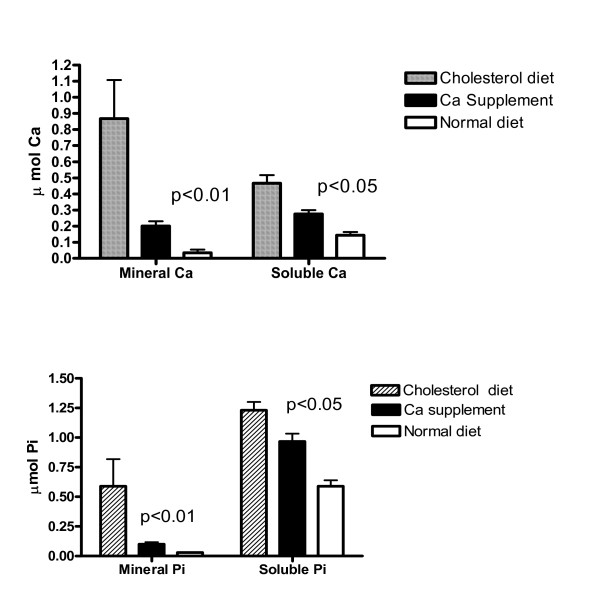
The inhibitory effect of calcium supplementation for 4 months on the dietary cholesterol-induced accumulation of mineral and soluble Ca/phosphate in aortas. The mineral content was expressed as the levels of the Ca (upper panel) and phosphate (lower panel) in 1% acetic acid-soluble fractions of 800 × g precipitates obtained from collagenase digestion of 1-cm aortas [8]. The soluble forms of Ca and phosphate were expressed as the Ca and phosphate content in the supernatant fractions, which were obtained by ultracentrifugation of the collagenase digests at 250,000 × g for 30 min.

To determine whether Ca diets may alter the accumulation of Ca in aortas, thereby affecting aortic calcification, soluble Ca from aortas was measured. Soluble Ca fractions were obtained by centrifuging the collagenase digests of aorta fragments at 300,000 × g for 30 min to precipitate all subcellular organelles. Cholesterol diets for 4 months caused a significant increase in the concentrations of soluble Ca and Pi (Fig. 7). Both mineral and the soluble Ca and Pi were decreased by Ca supplementation (p < 0.05). In rabbits on Ca deficient food for 2 1/2 months, there was a small increase in soluble Ca in aortas (data not shown), consistent with only a 10% of the cross-sections being calcified (Fig. 2D).

In summary, the observations herein reveal that dietary Ca levels in atherogenic diets not only profoundly affect atherosclerosis but also influence aortic calcification and icterus associated with cholesterol supplementation. Although the effects of dietary Ca were remarkable, the precise mechanisms whereby dietary Ca exerts its effects on atherosclerosis and calcification would be difficult to elucidate due to the multiplicity of Ca roles in various signal pathways in target cells and tissues.

## Conclusion

Ca supplementation to an atherogenic diet inhibits atherosclerosis, aortic calcification, and icterus, whereas a Ca deficient-diet promotes them.

## Experimental procedures

### Adjustments of dietary Ca levels in rabbit chows supplemented with cholesterol

To minimize the variation in the development of atherosclerosis from cholesterol supplementation because of genetic contributions [11-13], only siblings of young male 4-month-old New Zealand White rabbits (weighed about 2.5 kg) from identical litters were used. The rabbits were divided into 3 groups according to the levels of Ca in an atherogenic diet containing 0.5% cholesterol and 2% peanut oil. The first group was fed the atherogenic diet containing a normal 1% of dietary Ca by weight. The second group received the same atherogenic diet in which the Ca content was increased to 3 %. The third group was given the atherogenic diet in which the Ca level was reduced to 0.5%, which is the minimal that the manufacturer was able to prepare due to intrinsic Ca ingredients in rabbit chows (Harlan Teklad, Wisconsin). Since approximately 3 to 5 males per litter are available, separate litters at different times were needed for a statistically paired analysis. The content of Ca and cholesterol in the diet was routinely assessed by dissolving pellets in 1% acetic acid and 100% pyridine, respectively.

### Estimation of lesions and mineralization in rabbit thoracic aortas

Lesions developed fully in the proximal arch of thoracic aortas after 4 months of cholesterol feeding and progressed toward the distal section [9,14]. At this stage, about 50% of the middle sections (2.5 cm from the arch joint) were covered with thickened intima. Thus, we selected the middle section as an index of atherosclerosis to allow optimal statistical analysis for assessing the effects of dietary Ca. To study the effect of dietary Ca on calcification, the proximal sections of the aortas were selected as an index of calcification since calcification occurred mostly in this region. Less than 15% of the mid sections of the aortas was calcified and therefore would be far too small for statistical comparisons. Accordingly, the selection of different locations of the aorta was necessary for statistical analysis of the two processes. To semi-quantify the lesions and mineralization, the cross sections were stained with H&E and alizarin red, respectively, photographed and the images were magnified for photo prints. The weight ratios of the magnified images of the thickened intima to the media from the prints were calculated for lesion quantification. Likewise, for mineralization estimation, the alizarin red stained images were separated from the non-stained area of proximal thickened intima and weighed. The weight ratios of the images of the calcified area to the thickened intima were calculated as an index of calcification.

### Icterus diagnosis

Icterus was estimated from the extent of yellowing of the skin, sclera, and mucous membranes along with gross evidence of hepatic lipidosis and/or biliary obstructions assessed by the veterinarians at the Animal Resource Support Center. A scale of 0–5 was given to assess the degree of icterus.

### Serum lipids and Ca determinations

Since both serum and plasma are routinely used in rabbit lipid research, blood samples were collected and allowed to coagulate at room temperature without the use of heparin or EDTA, which otherwise affects Ca determinations. The blood samples were centrifuged at 1800 rpm for 10 min and the sera were collected for lipid analyses. The samples were properly diluted to standard assay ranges. The LDL-C content was directly measured by a standard clinical procedure without the application of Friedewald equation, which requires HDL and triglyceride data inputs to estimate LDL-C. The direct measurement procedure is included in the National Cholesterol Education Program Working Group on Lipoprotein Measurements. The lipid profile was assayed using a Beckman Synchron automation system in a clinical laboratory. The data are means ± S.E. The *p *values from the Student's *t *test denote statistical significance of differences between experimental and control rabbits. Ca and Pi determinations were performed using a commercial Arsenazo III dye kit (Sigma Chemicals Inc.) and a standard phosphate-molybdate complex colorimetric procedure, respectively.

## Competing interests

The author(s) declare that they have no competing interests.

## Authors' contributions

Howard H.T. Hsu, Ph.D. was responsible for the experimental designs, data interpretations, and execution of experiments. Nathan Culley, D.V. M. played a major role in icterus determinations, welfare and sacrifice of the rabbits, and collections of serum and aortas.
